# Clinical significance of CD8‐positive lymphocytes on tumor cell clusters of ascites cell block in ovarian high‐grade serous carcinoma

**DOI:** 10.1002/cam4.4592

**Published:** 2022-02-08

**Authors:** Hideki Iwahashi, Morikazu Miyamoto, Tsubasa Ito, Jin Suminokura, Taira Hada, Hiroki Ishibashi, Soichiro Kakimoto, Hiroko Matsuura, Rie Suzuki, Shinya Minabe, Susumu Matsukuma, Hitoshi Tsuda, Masashi Takano

**Affiliations:** ^1^ Department of Obstetrics and Gynecology National Defense Medical College Hospital Tokorozawa Japan; ^2^ Department of Laboratory Medicine National Defense Medical College Hospital Tokorozawa Japan; ^3^ Department of Basic Pathology National Defense Medical College Hospital Tokorozawa Japan

**Keywords:** ascites cell block, CD8‐positive lymphocytes, high‐grade serous carcinoma, hybrid cell count, immunohistochemical staining

## Abstract

**Background:**

The clinical significance of CD8‐positive (CD8^+^) lymphocytes on tumor cell clusters of ascites cell blocks in patients with ovarian high‐grade serous carcinoma (HGSC) was investigated.

**Methods:**

Among HGSC patients who underwent surgery from January 2014 to December 2019, 38 patients with ascites cell block were selected. Using these cell blocks and primary ovarian tumor tissue, the presence of CD8^+^ lymphocytes and the expression of PD‐L1 were examined immunohistochemically. Tumor cell clusters were defined as cell clumps consisting of more than 10 malignant cells in cell block. Cases with at least one CD8^+^ lymphocyte in tumor cell cluster were defined as positive CD8^+^ lymphocytes (Group A); others were defined as negative CD8^+^ lymphocytes (Group B). The tumor tissue CD8^+^ lymphocytes were counted mechanically. Clinicopathological features were retrospectively compared between the two groups.

**Results:**

In total, 38 cases were identified: 25 (65.8%) in Group A and 13 (34.2%) in Group B. More cases in Group A were positive for CD4 (*p* < 0.01), PD‐L1 (*p* = 0.02), FoxP3 (*p* = 0.02) and had a higher number of CD8^+^ lymphocytes in the tissue (*p* = 0.03). Patients in Group A had better progression‐free survival (*p* < 0.01) and overall survival (*p* = 0.04). In multivariate analysis, Group A was an independent prognostic factor for both progression‐free survival (hazard ratio, 0.24; *p* < 0.01) and overall survival (hazard ratio, 0.21; *p* = 0.03).

**Conclusion:**

The presence of CD8^+^ lymphocytes in tumor cell clusters of ascites was associated with the status of immune reaction in the tissue and prognosis in patients with HGSC and might be useful information of the immune‐associated therapy.

## Graphical abstract

The presence of CD8‐positive (CD8^+^) lymphocytes on tumor cell clusters of ascites cell blocks in patients with ovarian high‐grade serous carcinoma was associated with the number of CD8‐positive (CD8^+^) lymphocytes and prognosis. This information might be useful for the status of immune reaction.

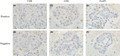

## 1 INTRODUCTION

Ovarian carcinoma is the leading death cause of patients with gynecologic malignancies, and its incidence has been increasing worldwide.^1^ Despite advanced treatments including the combination of maximum debulking surgery and chemotherapy, the patients' prognosis remains poor.^2^ The histological type, age of the patient, stage of cancer (International Federation of Gynecology and Obstetrics [FIGO] stage), and residual tumor size after debulking surgery are well‐known prognostic factors of ovarian carcinoma.^2^, ^3^, ^4^, ^5^, ^6^ Among the histological types, high‐grade serous carcinoma (HGSC) is the most prevalent, is frequently diagnosed in advanced stages, and shows a better response to platinum‐based chemotherapy.^7^

CD8‐positive (CD8^+^) lymphocytes have antitumor effect to directly attack the tumor cells. Many recent studies have demonstrated that CD8^+^ lymphocytes, one of tumor‐infiltrating lymphocytes (TILs), in the tissue were the predictive prognostic factor in several carcinomas.^8^, ^9^, ^10^, ^11^, ^12^, ^13^ Similarly, the number of CD8^+^ lymphocytes in the tissue was a predictive factor for the prognosis of patients with HGSC.^14^, ^15^, ^16^ Furthermore, several researchers have suggested that increasing the number of CD8^+^ lymphocytes in ascites was a better prognostic factor in ovarian carcinoma.^17^, ^18^, ^19^ These findings indicated that the presence of CD8^+^ lymphocytes in tissues and ascites could be predictive biomarkers of prognosis in patients with HGSC.

On the other hand, there are other factors related to tumor immunity such as CD4^+^ lymphocytes which recognize major histocompatibility complex class II cancer antigen of dendritic cells and induce several immune reactions.^20^ Conversely, forkhead box P3^+^ (FoxP3^+^) lymphocytes which play a suppressive role in tumor immunity^21^, ^22^, ^23^ and programmed death ligand 1 (PD‐L1) which is involved in the immune escape mechanism are also an important factor of tumor immunity.^24^, ^25^ Also, these immune reactive factors were associated with the prognosis of patients with ovarian cancer.^21^, ^22^, ^23^, ^24^, ^25^

In recent years, the development of immune checkpoint inhibitors and the search for its biomarkers has been conducted. Tumors with mismatch repair (MMR) deficiency upregulated immune suppressive factors such as PD‐L1, which was associated with escape from tumor immunity such as CD8^+^ lymphocytes.^24^, ^25^ Therefore, MMR status is useful as the biomarker to predict the efficacy of the immune checkpoint inhibitors for colorectal and uterine corpus carcinoma.^24^, ^26^

Ascites cell block is a useful specimen for morphological analysis, immunohistochemical (IHC) analysis, and repeated examination with the same specimen and method based on paraffin‐embedded tissue specimens.^27^, ^28^ This study aimed to examine the clinical significances of CD8^+^ lymphocytes evaluated by ascites cell block and the relationships between these CD8^+^ lymphocytes and other factors associated with immune response.

## 2 MATERIALS AND METHODS

### 2.1 Patient selection

Patients with HGSC who underwent primary debulking surgery followed by the combination chemotherapy of paclitaxel and carboplatin chemotherapy as adjuvant chemotherapy, without neoadjuvant chemotherapy, at our hospital between January 2014 and December 2019 were identified retrospectively for this study. Among them, patients with ascites cell blocks made using ascites collected during primary surgery were included in our study. Patients with those who received only peritoneal lavage cytology were excluded. Clinical and surgical information was obtained from medical and surgical records.

### 2.2 Immunohistochemical stain for ascites cell block and tumor tissue

Ascites collection, ascites cell block formation, and IHC staining were performed as previously reported.^29^, ^30^ The primary antibodies used are shown in Table 1. Ascites cell blocks were stained with all antibodies, and the tumor tissue was stained with only CD8 antibody. As a negative control, tissue slides incubated without primary antibodies were used.

**TABLE 1:** Primary antibodies

Molecule	Type	Antibody Clone/Code	Manufacturer	Dilution	Localization	Control tissue	Antigen retrieval
CD8	Monoclonal (Mouse)	C8/144B	Dako	×50	Membrane	Tonsil	Citrate
CD4	Monoclonal (Mouse)	SP35	Abcam	×50	Membrane	Spleen	EDTA
FoxP3	Monoclonal (Mouse)	236A/E7	Abcam	×100	Nucleus	Tonsil	EDTA
MLH1	Monoclonal (Mouse)	ES05	Dako	×100	Nucleus	Appendix	EDTA
MSH2	Monoclonal (Mouse)	FE11	Dako	×400	Nucleus	Appendix	EDTA
MSH6	Monoclonal (Rabbit)	44	Biocare Medical	×200	Nucleus	Colon	EDTA
PMS2	Monoclonal (Rabbit)	EP51	Dako	×10	Nucleus	Appendix	EDTA
PD‐L1	Monoclonal (Rabbit)	EPR19759	Abcam	×250	Membrane and endomembrane	Placenta	Citrate

Abbreviations: EDTA, ethylenediaminetetraacetic acid; FoxP3, forkhead box P3; MLH1, MutL homolog 1; MSH2, MutS homolog 2; MSH6, MutS homolog 6; PD‐L1, programmed death ligand 1; PMS2, postmeiotic segregation increased 2.

### 2.3 Immunohistochemistry interpretation using ascites cell block

**FIGURE 1:**
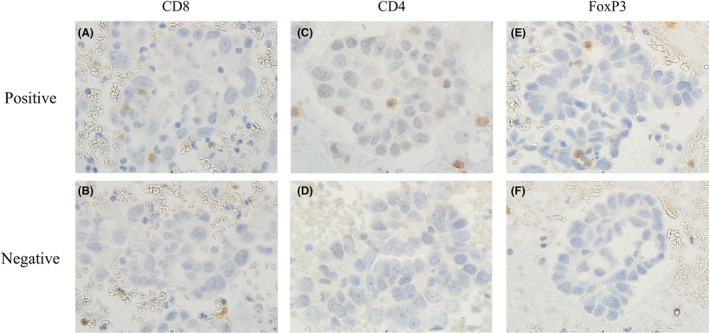
Representative image of ascites cell block IHC for CD8, CD4, and FoxP3. CD8^+^ lymphocytes were observed on the tumor cell cluster (A). Such cases were defined as Group A. CD8^+^ lymphocytes were observed around the tumor cell cluster, but no CD8^+^ lymphocytes were observed on the tumor cell cluster (B). Such cases were defined as Group B. Similarly, CD4^+^ lymphocytes and FoxP3^+^ lymphocytes were categorized into positive and negative as well as CD8^+^ lymphocyte definition. CD4^+^ lymphocytes were not observed on the tumor cell cluster of all cases (C). FoxP3^+^ lymphocytes were observed around the tumor cell cluster (D), but no FoxP3^+^ lymphocytes were observed on the tumor cell cluster (E). Original magnification: ×1000

IHC analysis was performed under a light microscope without clinical information. In the evaluation of ascites cell blocks, tumor cell clusters were defined as cell clumps that consisted of more than 10 malignant cells. Cases with less than 30 tumor cell clusters in one slide were excluded. The presence of at least one CD8^+^ lymphocyte on the tumor cell cluster was defined as positive CD8^+^ lymphocytes (Figure 1A, ×1000), and the others were defined as negative CD8^+^ lymphocytes (Figure 1B, ×1000). Patients with positive CD8^+^ lymphocytes were defined as Group A. Cases with negative CD8^+^ lymphocytes were defined as Group B. Additionally, the presence of at least one CD4^+^ lymphocyte on a tumor cell cluster was defined as positive CD4^+^ lymphocytes and the others were defined as negative CD4^+^ lymphocytes (Figure 1C, ×1000). Additionally, at least one FoxP3^+^ lymphocyte on a tumor cell cluster was defined as positive FoxP3^+^ lymphocytes (Figure 1D, ×1000), and the others were defined as negative FoxP3^+^ lymphocytes (Figure 1E, ×1000).

**FIGURE 2:**
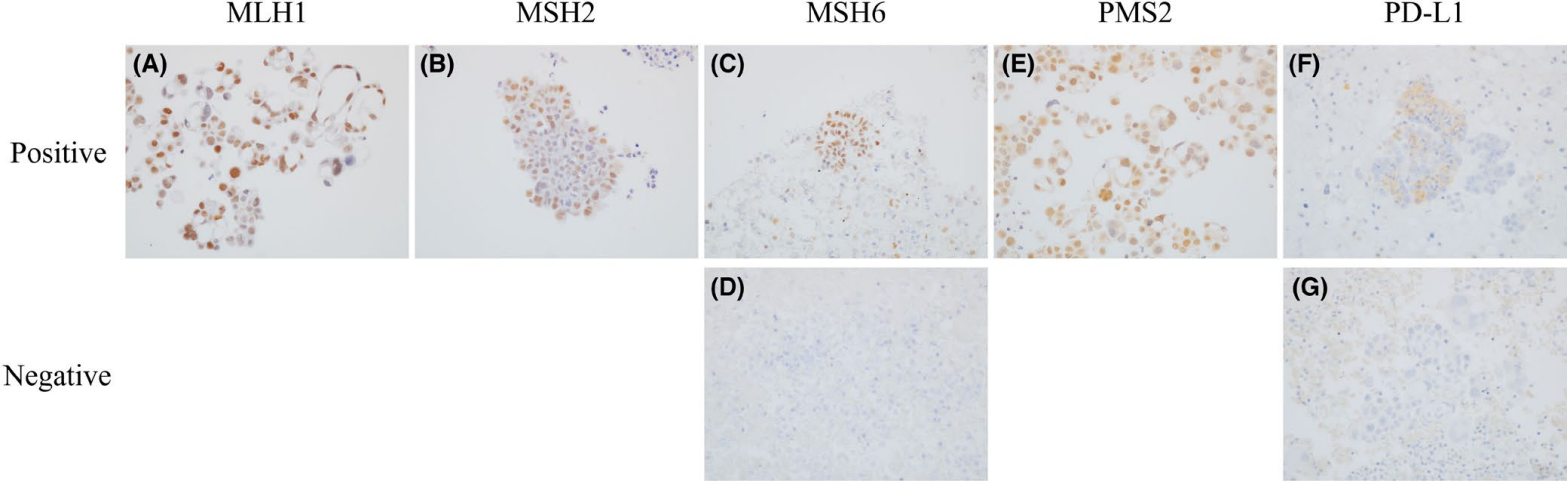
Representative image of positive and negative cases for MLH1, MSH2, MSH6, PMS2, and PD‐L1 of the ascites cell block. MLH1 (A), MSH2 (B), MSH6 (C), and PMS2 (E) were diffusely positive in the tumor cell cluster. Only one case was considered as negative for MSH6 (D). PD‐L1 was expressed on the cellular membrane so as to surround the tumor cell cluster in positive cases (F), and not in negative cases (G). Original magnification: ×400. MLH1, MutL homolog 1; MSH2, MutS homolog 2; MSH6, MutS homolog 6; PMS2, postmeiotic segregation increased 2; PD‐L1, programmed death‐ligand 1

Additionally, MutL homolog 1 (MLH1), MutS homolog 2 (MSH2), MutS homolog 6 (MSH6), and postmeiotic segregation increased 2 (PMS2) were stained as mismatch repair (MMR)‐related proteins. Interpretation of MMR‐related proteins and MMR status was performed as previously reported.^26^ Cases were defined as positive if any stained nuclei with immunoreactive intensity stronger than or equal to positive controls were observed, and the others were defined as negative (Figure 2A–E, ×400). Cases with all positive for MLH1, MSH2, MSH6, and PMS2 were defined as MMR‐retained, and the others were defined as MMR deficient. Cases with PD‐L1 immunoreactivity on more than 1% of tumor cells consisting of tumor cell clusters were defined as positive PD‐L1 expression (Figure 2F, ×400), and the others were defined as negative PD‐L1 expression (Figure 2G, ×400).

### 2.4 Immunohistochemistry interpretation using tissue

**FIGURE 3:**
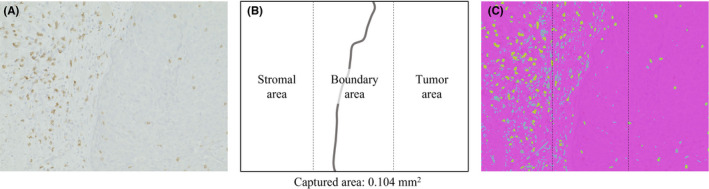
Representative image of hybrid cell count of CD8^+^ lymphocytes in tumor tissue. Nine areas were selected and photographed so that the tumor stroma, borderline area, and stroma were approximately 1:1:1 (A, B) by ×400 magnification. CD8^+^ lymphocytes at the same site were marked and a hybrid cell count was performed with BZ‐X800 (Keyence) (C)

The tissue slide evaluated by IHC was made from primary lesions not metastatic lesions. The counting method for CD8^+^ lymphocytes in tissue was as follows: in a single slide including the primary tumor, nine areas with many CD8^+^ lymphocytes at invasive front were chosen (Figure 3A). The images were divided into three equal parts: tissue, boundary, and stroma parts (Figure 3B, ×200). CD8^+^ lymphocytes within the area (0.104 mm^2^ in each area) were detected by fluorescence immunostaining, and the number of cells was calculated using Hybrid Cell Count software (BZ‐X800; Keyence, Figure 3C), as previously reported.^31^ The maximum number of CD8^+^ lymphocytes among the nine areas measured by the hybrid cell count was defined as the CD8^+^ lymphocyte count in the tissue.

### 2.5 The number of CD8^+^ lymphocytes in cell block

The number of CD8^+^ lymphocytes which did not form cell cluster in the background of cell block was counted as similar method to count the number of CD8^+^ lymphocyte in the tissue. Briefly, nine sites without tumor cell clusters were randomly selected. The number of CD8^+^ and FoxP3^+^ lymphocytes were counted using Hybrid Cell Count software (BZ‐X800; Keyence).

### 2.6 Statistical analysis

JMP® Pro ver. 14.0.0 (SAS Institution Inc.) was used for statistical analysis. The chi‐squared test or Fisher's exact test were used to compare characteristics. The disease stage was determined according to the 2014 FIGO staging classification.^32^ Residual tumors <1 cm were defined as optimal surgery, and residual tumors ≥1 cm were defined as suboptimal surgery at the point of primary debulking surgery. The evaluation of the response of chemotherapy of the patients with measurable residual diseases at primary surgery was performed by The Response Evaluation Criteria in Solid Tumors (RECIST) version 1.1.^33^ The response was described as best response after primary surgery. Progression‐free survival (PFS) was defined as the time from initial diagnosis of the disease to the diagnosis of progression or death. Overall survival (OS) was defined as the time from the initial diagnosis of the disease to death or the date of the last follow‐up contact. Receiver operating characteristic (ROC) curve analysis was performed using CD8^+^ lymphocyte counts in tissue, Group A, and Group B, with the cut‐off value set. Kaplan–Meier survival curves for PFS and OS were compared using log‐rank tests. The Cox regression hazard model was used for the univariate and multivariate analyses of PFS and OS. The multivariate analysis was performed by the variables that were statistically significant in the univariate analysis. Statistical significance was defined as a *p‐*value of <0.05. The clinical data of this study are available to the extent that their use does not infringe patient privacy.

## 3 RESULTS

**FIGURE 4:**
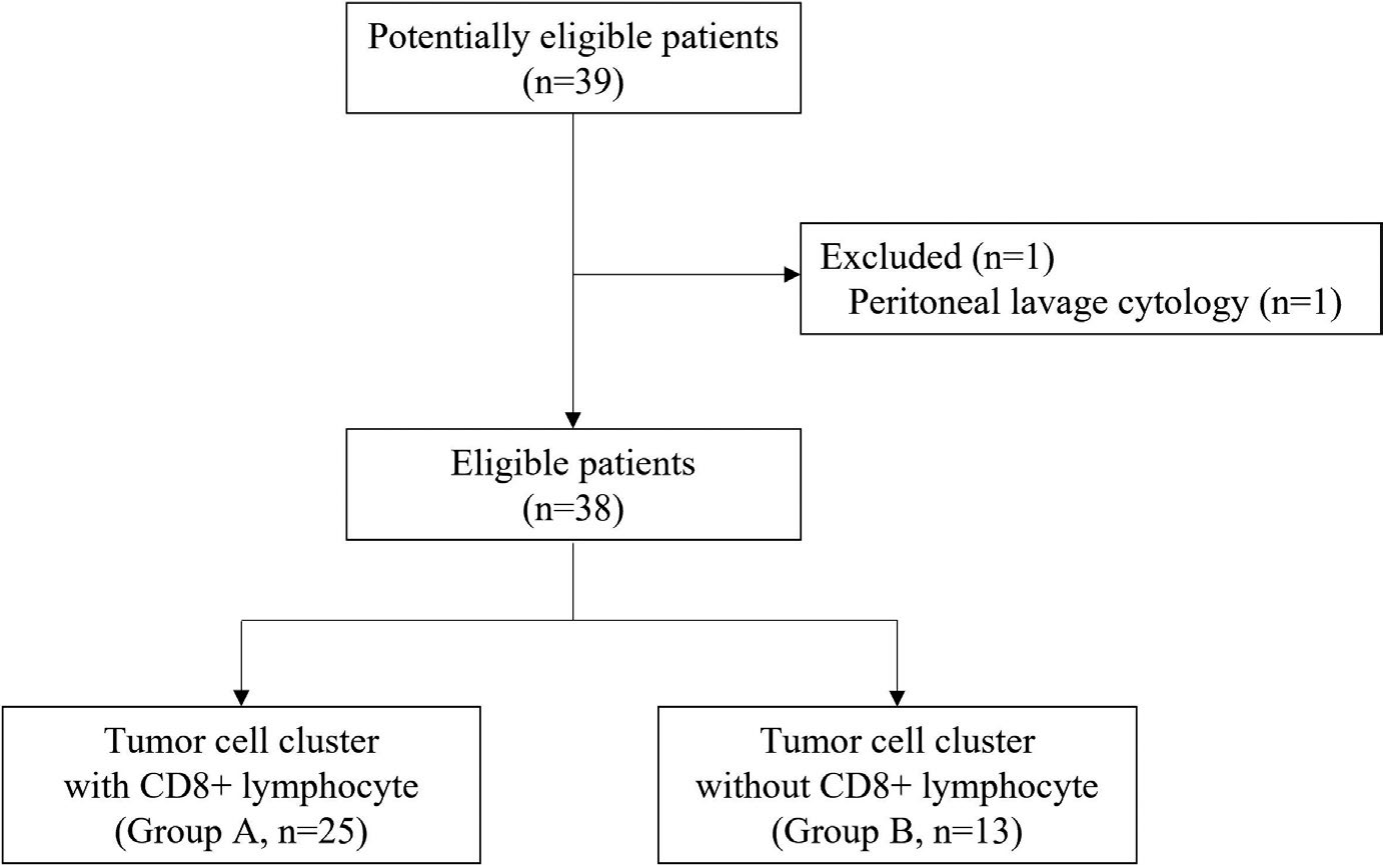
The diagram of our study. Cell block was made from 39 cases with high‐grade serous carcinoma. One case was excluded due to peritoneal lavage cytology. Our study included 38 cases; 25 cases were classified into Group A, and 13 cases were Group B

The diagram of our study is shown in Figure 4. Cell block was made from 39 cases with HGSC. One case was excluded due to peritoneal lavage cytology. Data from 38 cases with HGSC were included in our study. CD4^+^, CD8^+^, and FoxP3^+^ lymphocytes were observed in the background of cell block specimen. Also, PD‐L1 was expressed in many tumor cells which did not form clusters.

There were 25 (65.8%) cases in Group A and 13 (34.2%) in Group B. Patient characteristics are shown in Table 2. There were no significant differences in age (*p* = 0.70), FIGO stage (*p* > 0.99), residual tumor status (*p* > 0.99), lymph node metastasis (*p* > 0.99), adjuvant chemotherapy (*p* > 0.99), and clinical response (*p* = 0.49) between the two groups. More cases with positive CD4^+^ lymphocyte (*p* < 0.01), positive PD‐L1 expression (*p* = 0.02), and positive FoxP3^+^ lymphocytes (*p* = 0.02) were observed in Group A than in Group B.

**TABLE 2:** Patient characteristics

	Group A (*n* = 25)	Group B (*n* = 13)	*p*‐value
Age (median)	66 (39–84)	64 (29–82)	0.70
FIGO stage (%)			
I, II	1 (4.0)	0 (0.0)	>0.99
III, IV	24 (96.0)	13 (100)	
Residual tumor status (%)			
Optimal	12 (48.0)	6 (46.2)	>0.99
Suboptimal	13 (52.0)	7 (53.8)	
Lymph node metastasis (%)			
Present	9 (36.0)	4 (30.8)	>0.99
Absent	14 (56.0)	8 (61.5)	
No collection	2 (8.0)	1 (7.7)	
Adjuvant chemotherapy (%)			
Done	25 (100)	13 (100)	>0.99
Not done	0 (0.0)	0 (0.0)	
Response of the chemotherapy (%)			
CR/PR	14 (56.0)	12 (92.3)	0.49
SD/PD	2 (8.0)	0 (0.0)	
CD4^+^ lymphocyte on tumor cell cluster (%)			
Positive	21 (84.0)	3 (23.1)	<0.01
Negative	4 (16.0)	10 (76.9)	
PD‐L1 expression (%)			
Positive	18 (72.0)	4 (30.8)	0.02
Negative	7 (28.0)	9 (69.2)	
FoxP3^+^ lymphocyte on tumor cell cluster (%)			
Positive	17 (68.0)	3 (23.1)	0.02
Negative	8 (32.0)	10 (76.9)	
MMR status (%)			
Retained	24 (96.0)	13 (100)	>0.99
Deficient	1 (4.0)	0 (0.0)	
CD8^+^ lymphocyte count at tissue (%)			
≥Cut‐off value	23 (92.0)	8 (61.5)	0.03
<Cut‐off value	2 (8.0)	5 (38.5)	

*Note*: Cases with at least one CD8^+^ lymphocyte on tumor cell cluster were defined as Group A; the others were defined as Group B. Tumor CD8^+^ lymphocyte counts were classified into higher and lower groups according to ROC curve analysis for CD8^+^ lymphocyte count using hybrid cell count. The response was evaluated for the patients with measurable disease at primary surgery. Abbreviations: CR, complete response; FIGO, International Federation of Gynecology and Obstetrics; FoxP3, forkhead box P3; MMR, mismatch repair; PD, progressive disease; PR, partial response; PD‐L1, programmed death ligand 1; SD, stable disease.

**FIGURE 5:**
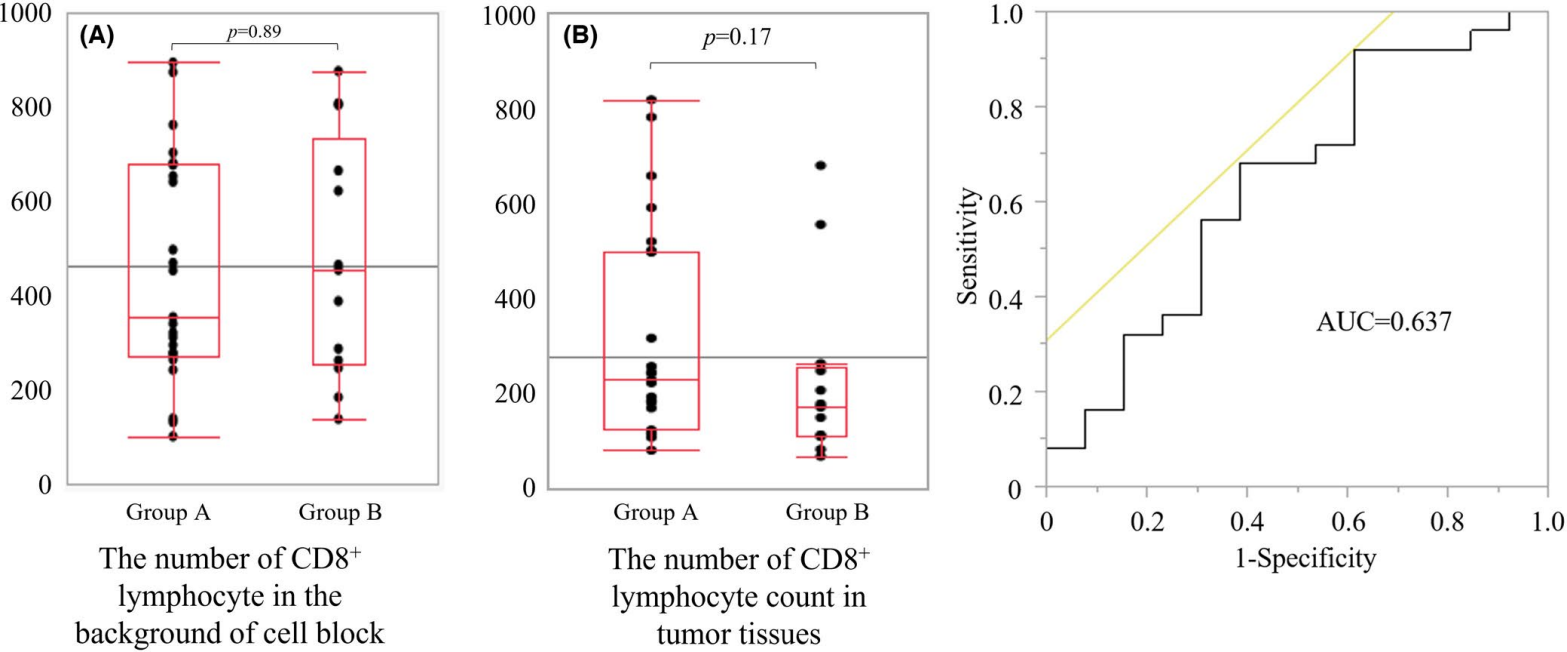
The comparison of the number CD8^+^ lymphocytes in the background of cell block (A) and tumor tissue (B) between Group A and B and the ROC curves for the number of CD8^+^ lymphocytes in tumor tissues (C). The median count of CD8^+^ lymphocytes in the background was 354 (lower quartile 269, upper quartile 679) for Group A, and 454 (lower quartile 254, upper quartile 734) for Group B (A). There was no statistical significance between Group A and B (*p =* 0.89). The median count of CD8^+^ lymphocytes in tumor tissue was 219 (lower quartile 100, upper quartile 530) for Group A, and 153 (lower quartile 84, upper quartile 250) for Group B. There was no statistical significance between Group A and B (*p* = 0.17). ROC curves for tumor tissue CD8^+^ lymphocyte and CD8^+^ lymphocyte status for each group (B). The cutoff value for the CD8^+^ lymphocyte counts was calculated to be 88

The comparison of the number of CD8^+^ lymphocytes in the background of cell blocks and tumor tissues and the ROC curve was shown in Figure 5. Median CD8^+^ lymphocytes count of Group A and Group B in the background of cell blocks were 354 (interquartile range [IQR] 269–679) and 454 (IQR 254–734), respectively (Figure 5A). There were no statistical significances between two groups (*p* = 0.89). The median count of CD8^+^ lymphocytes in tumor tissues of Group A and Group B were 219 (IQR 100–530) and 153 (IQR 84–250), respectively (Figure 5B). There were no statistical significances between two groups (*p* = 0.17). Figure 5C showed the combined ROC curve of Group A or Group B and the number of CD8^+^ lymphocyte in tumor tissues. The area under the curve was 0.637. With the cut‐off value of CD8^+^ lymphocyte count of tissue of 88 counts, the sensitivity and specificity were 92.0% and 54.8%, respectively (Figure 5C). In Group A, there were more cases with ≥ cut‐off value of CD8^+^ lymphocyte count in the tissue (*p* = 0.03).

**FIGURE 6:**
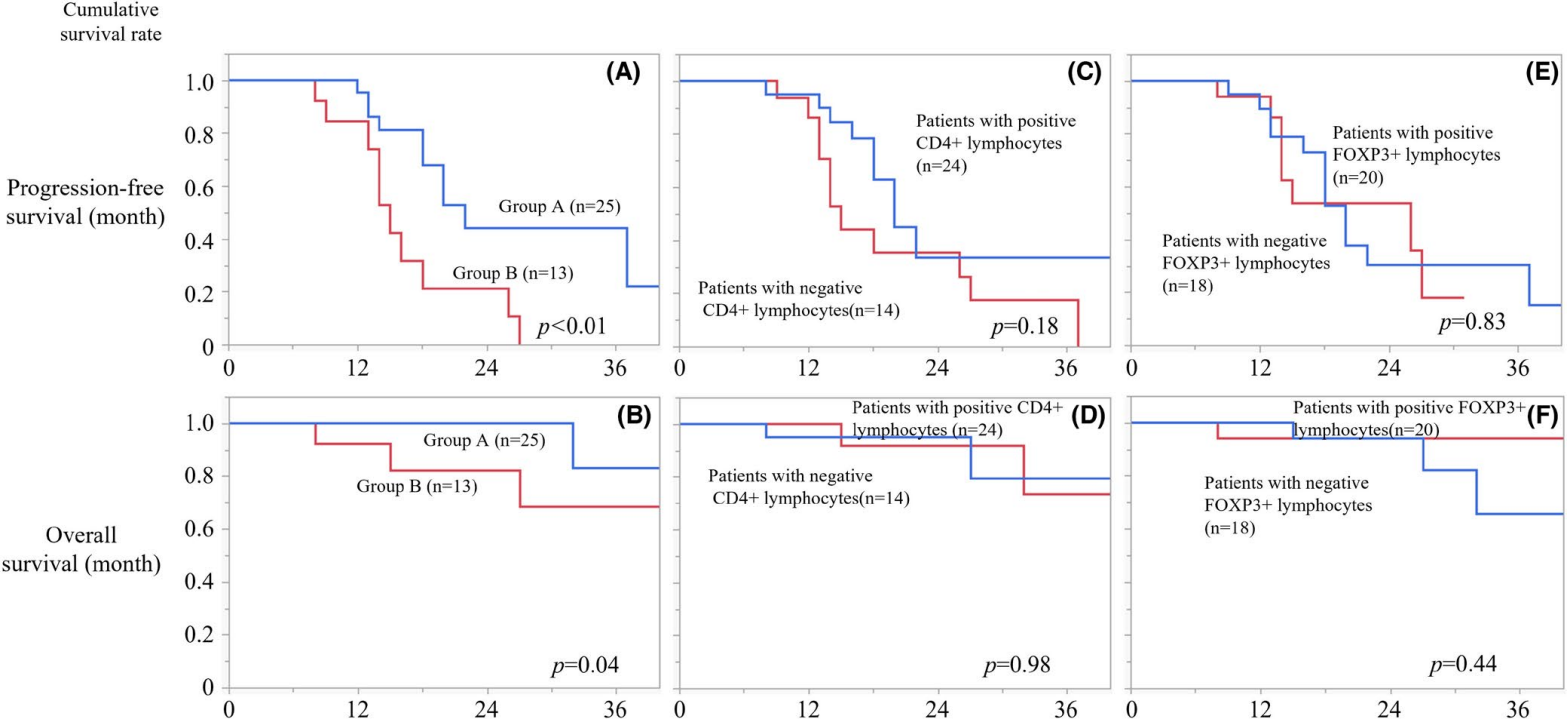
Progression‐free survival (PFS) and overall survival (OS) according to status CD8^+^, CD4^+^, FOXP3^+^ lymphocyte in cell block. PFS (A, *p* < 0.01) and OS (B, *p* = 0.04) of Group A defined as patients with positive CD8^+^ lymphocytes were better than Group B defined as patients with negative CD8^+^ lymphocytes. There were no statistical significances of PFS (C, *p* = 0.18) and OS (D, *p* = 0.98) according to CD4^+^ lymphocyte. Similarly, FOXP3^+^ lymphocyte was not the prognostic factor of PFS (E, *p* = 0.83) and OS (F, *p* = 0.44)

Of all the cases, only one case in Group A was determined to be MMR‐deficient (2.6%), and there were no significant differences for MMR status between the two groups. Patients in Group A had better PFS (Figure 6A, *p* < 0.01) and OS (Figure 6B, *p* = 0.04). On the other hand, there were no significances of PFS (Figure 6C, *p* = 0.18) and OS (Figure 6D, *p* = 0.98) between patients with positive CD4^+^ lymphocyte and those with negative CD4^+^ lymphocyte. Also, FOXP3+ lymphocyte did not affect PFS (Figure 6E, *p* = 0.83) and OS (Figure 6F, *p* = 0.44).

Multivariate analyses for PFS and OS revealed that Group A was an independent prognostic factor for PFS (hazard ratio 0.07; *p* < 0.01) and OS (hazard ratio 0.05; *p* = 0.04) (Table 3).

**TABLE 3:** Variables predictive of progression‐free and overall survival at univariate and multivariate analysis

Variables	Progression‐free survival						Overall survival					
	Univariate analysis			Multivariate analysis			Univariate analysis			Multivariate analysis		
	HR	(95% CI)	*p*‐value	HR	(95% CI)	*p‐*value	HR	(95% CI)	*p‐*value	HR	(95% CI)	*p‐*value
Age												
<65 versus ≥65	0.74	(0.28–1.84)	0.53				0.42	(0.29–3.68)	0.51			
FIGO stage												
III versus IV	0.43	(0.16–1.26)	0.12				0.51	(0.12–3.94)	0.47			
Residual tumor status												
Optimal versus Suboptimal	0.24	(0.08–0.72)	0.01	0.17	(0.05–0.53)	<0.01	0.32	(0.12–0.93)	0.03	0.12	(0.08–0.94)	0.03
Patient group												
Group A versus Group B	0.25	(0.14–0.88)	0.03	0.24	(0.10–0.65)	<0.01	0.26	(0.08–0.96)	0.04	0.21	(0.11–0.91)	0.03
PD‐L1 expression												
Positive versus Negative	0.87	(0.34–2.23)	0.78				1.36	(0.13–13.5)	0.79			
FoxP3^+^ lymphocyte												
Positive versus Negative	0.90	(0.36–2.29)	0.84				2.37	(0.25–22.8)	0.46			
CD4^+^ lymphocyte												
Positive versus Negative	0.55	(0.22–1.36)	0.19				1.02	(0.14–7.39)	0.98			

*Note*: Cases with at least one CD8^+^ lymphocyte in the tumor cell cluster were defined as Group A and the others were defined as Group B.

Abbreviations: CI, confidence interval; FIGO, International Federation of Gynecology and Obstetrics; FoxP3, forkhead box P3; HR, hazard ratio; PD‐L1, programmed death ligand 1.

## 4 DISCUSSION

Our study showed that the presence of CD8^+^ lymphocytes in tumor cell clusters in ascites cell blocks was related to PD‐L1 expression and FoxP3^+^ lymphocytes of tumor cell clusters in the ascites cell block, high CD8^+^ lymphocyte count at the tumor tissue, and was a good prognostic factor for patients with HGSC.

Previous studies have shown that the frequency of PD‐L1 expression in tumor tissues of HGSC ranges from 19.5% to 69.3%.^15^, ^34^, ^35^ In our study, there was positive PD‐L1 expression in the ascites cell block tumor cells in 22 of 37 (57.9%) cases. Therefore, PD‐L1 expression might be evaluated in ascites cell blocks similar to tumor tissues.

Tumor infiltration and attack by CD8^+^ lymphocytes induce PD‐L1 expression on the surface of tumor cells which lead adaptive resistance to recognition from antitumor immunity.^15^, ^36^ Similarly, FoxP3^+^ lymphocytes, which act as immune suppressive regulators, emerge in response to immune reactions such as CD8^+^ lymphocytes.^37^ Thus, a high number of FoxP3^+^ lymphocytes are associated with the antitumor activity of CD8^+^ lymphocytes.^21^ Therefore, we assumed that the reason that Group A had more cases with positive PD‐L1 expression or positive CD4^+^ and FoxP3^+^ lymphocytes on tumor cell clusters was because of the immune reaction system as mentioned above occurred.

In previous studies, high numbers of CD8^+^ lymphocytes in the tissue were good prognostic factors for patients with HGSC.^14^, ^38^, ^39^, ^40^, ^41^, ^42^ Although CD8^+^ lymphocytes almost invariably were the better prognostic factor, evaluation methods of CD8^+^ lymphocytes was different among several reports. Some reports evaluated stromal CD8^+^ lymphocytes,^38^, ^39^ and others evaluated the tumor invasive fronts,^14^, ^40^ or both the stroma and the tumor invasive fronts.^41^, ^42^ However, CD8^+^ lymphocytes emerging at any lesions in tissue was the important factor because CD8^+^ lymphocytes were related with better prognosis. Therefore, in our study, CD8^+^ lymphocytes at invasive front and stroma in tissue were evaluated. As results, Group A was associated with the high number of CD8^+^ lymphocytes in tissue. Thus, CD8^+^ lymphocytes at tumor cell cluster on cell block might be useful to predict the status of CD8^+^ lymphocytes in tissue.

The frequency of MMR deficiency in HGSC ranges from 0% to 13.4%.^25^, ^43^ In our study, only one (2.6%) case had MMR deficient. This frequency was relatively low compared to that in previous reports. In addition, although an association between MMR deficiency and TILs has been reported,^10^ it was not observed in our study. Further research to examine this problem is needed because our study included only a small number of cases.

The limitations of our study include the small number of cases analyzed and the single‐institutional retrospective analysis. Although it was interesting, we did not perform the prognostic analysis of PD‐L1 and FoxP3 using tissue samples because we should set the new criteria to judge immunochemical analysis and the study was too complexed to understand. We will plan the future research about this association in tissue. Also, our study did not examine the other cells associated with tumor immunity such as NK cells. However, the presence of CD8^+^ on tumor cell clusters was associated with the number of CD8^+^ lymphocytes in the tissue, which resulted in a better prognosis for patients with HGSC. The CD8+ biomarker in this method may be a lead to a more effective prognosis of ovarian HGSC.

## 5 CONCLUSION

The presence of CD8^+^ lymphocytes on tumor cell clusters of ascites cell blocks may demonstrate a better prognosis in patients with ovarian high‐grade serous carcinoma.

## CONFLICT OF INTEREST

All authors have no conflicts of interest to disclose.

## AUTHOR CONTRIBUTIONS

Hideki Iwahashi, Morikazu Miyamoto, and Masashi Takano carried out protocol/project development. Hideki Iwahashi, Morikazu Miyamoto, Tsubasa Ito, Jin Suminokura, Taira Hada, Hiroki Ishibashi, Soichiro Kakimoto, Hiroko Matsuura, and Rie Suzuki were involved in data collection and management. Hideki Iwahashi, Morikazu Miyamoto, Shinya Minabe, Susumu Matsukuma, and Hitoshi Tsuda carried out data analysis. Hideki Iwahashi, Morikazu Miyamoto, and Masashi Takano were involved in writing and editing.

## ETHICAL APPROVAL STATEMENT

This study was approved by the institutional review board of National Defense Medical College (Approval No. 3078). The study did not require written informed consent for retrospective analysis.

## Data Availability

The data analyzed in the current study are available from the corresponding author on reasonable request.
